# Crystal Structure of the Japanese Encephalitis Virus Capsid Protein

**DOI:** 10.3390/v11070623

**Published:** 2019-07-06

**Authors:** Thanalai Poonsiri, Gareth S. A. Wright, Tom Solomon, Svetlana V. Antonyuk

**Affiliations:** 1 Molecular Biophysics Group, Institute of Integrative Biology, Faculty of Health and Life Sciences, University of Liverpool, L69 7ZB Liverpool, UK; 2 Health Protection Research Unit on Emerging and Zoonotic Infections, Institute of Infection and Global Health, University of Liverpool, L69 7BE Liverpool, UK; 3 Walton Centre NHS Foundation Trust, L9 7LJ Liverpool, UK

**Keywords:** flavivirus, core protein, x-ray crystallography, homodimer

## Abstract

Japanese encephalitis (JE) is inflammation and swelling of the brain caused by the JE virus (JEV), a mosquito-borne member of the Flavivirus family. There are around 68,000 JE cases worldwide each year, many of which result in permanent brain damage and death. There is no specific treatment for JE. Here we present the crystal structure of the JEV capsid protein, a potential drug target, at 1.98 Å, and compare it to other flavivirus capsid proteins. The JEV capsid has a helical secondary structure (α helixes 1–4) and a similar protein fold to the dengue virus (DENV), the West Nile virus (WNV), and the Zika virus (ZIKV) capsid proteins. It forms a homodimer by antiparallel pairing with another subunit (‘) through α-helix 1-1’, 2-2’, and 4-4’ interactions. This dimeric form is believed to be the building block of the nucleocapsid. The flexibility of the N-terminal α helix-1 allows the formation of closed and open conformations with possible functional importance. The basic C-terminal pairing of α4-4’ forms a coiled-coil-like structure, indicating possible nucleic acid binding functionality. However, a comparison with other nucleic acid interacting domains indicates that homodimerization would preclude binding. This is the first JEV capsid protein to be described and is an addition to the structural biology of the Flavivirus.

## 1. Introduction

The Japanese encephalitis virus is a flavivirus transmitted by Culex mosquitoes and is closely related to other serious emerging viruses, including the dengue virus (DENV), the West Nile virus (WNV), and the Zika virus (ZIKV). In severe cases, the Japanese encephalitis virus (JEV) causes inflammation and swelling of the brain, with 10–20% of patients dying and over half the survivors left with brain damage [1]. The JEV is a major cause of viral encephalitis in Asia. Its positive-sense single strand RNA genome (~11 kb) codes for three structural proteins: Capsid (C), membrane (prM/M), and envelope protein (E), and seven non-structural (NS) proteins: NS1, NS2A, NS2B, NS3, NS4, NS4B, and NS5, which are translated as a precursor polyprotein. The external shell of the virus is formed by the endoplasmic reticulum (ER)-derived lipid bilayers inserted with E and M proteins [2,3,4,5]. Receptor binding by viral E protein plays a role in host cell invasion by fusing to the endosomal membrane to release the nucleocapsid into the cytoplasm. The nucleocapsid is composed of multiple copies of the capsid protein, enclosing the single-stranded RNA genome. Dissociation of the capsid releases the viral RNA, which templates viral protein translation using the host cell machinery. The newly synthesized NS proteins form the replication complex in vesicle packets, generated from the ER [6]. Once the viral RNA is synthesized, it is enclosed within the capsid, which buds into the ER to obtain the host cell lipid bilayer together with the E and prM proteins. Cleavage of prM to form the M protein in the Golgi body by the enzyme furin produces the mature virion primed for release from the cell to begin a new infection cycle.

C proteins have roles, including, but not limited to, nucleocapsid dissociation and assembly. Many studies have revealed the crucial functions of the C protein in the flavivirus life cycle [7,8]. Mutation of C protein N-terminal residues has been demonstrated to impair the DENV particle formation [9,10]. The DENV and WNV C proteins were shown to act as RNA chaperones [11,12], and the RNA binding sites were mapped to positively charged residues in the WNV C protein N and C termini [13]. The capsid protein has wide subcellular distribution, including cytoplasm, ER, lipid droplets (LDs), and the nucleoli of infected cells [14,15,16]. However, the rationale for this remains unclear. Mutagenesis studies identified a hydrophobic patch in the helix-2 region as important for viral replication [17] and membrane interaction [18,19]. Moreover, the capsid protein was reported to interact with several host proteins, including the death-associated protein-6 (DAXX), which may induce apoptosis, and core histones that mediate transcription through nucleosome disruption [7].

JEV E [5,20,21], NS3 (protease-helicase) [22,23], and NS5 (methyltransferase-polymerase) [24,25] proteins are well described in structure and function. As the E protein is important for viral entry and the latter two are crucial enzymes for viral propagation, they are the main targets for drug development. Many small-interfering RNAs, small-molecules, peptide inhibitors, and nucleoside analogues targeting E, NS3, or NS5 proteins have been tested, along with antagonistic molecules, immune modulators, and host enzyme inhibitors, using in vitro and animal models. However, none have proceeded to or succeeded in clinical trials [26,27]. With mounting evidence suggesting significant roles for the C protein in the flavivirus life cycle, this multifunctional protein may be a target for new antiviral development. Capsid protein is already considered an antiviral therapy target in many non-flavivirus related diseases, such as the human immunodeficiency virus (HIV), the hepatitis B virus, and the enterovirus [28,29,30]. With the recent addition of our structural characterization of NS1 [31], the JEV protein structural study is only missing the C protein and non-structural transmembrane protein structures. Here, we present the JEV capsid protein structure at 1.98 Å resolution. The JEV capsid protein has a conserved dimeric protein fold when compared with the DENV, WNV, and ZIKV C proteins. Important protein properties, including dimerization of the nucleocapsid building block and functionally important α-helix-1 flexibility, are suggested by this structure.

## 2. Materials and Methods

### 2.1. Plasmid Construction

The JEV capsid DNA (nucleotide residue 1–315) lacking the coding sequence for the hydrophobic C-terminal, which ends at the natural NS3 protease cleavage site (102 QNKR↓GGNE 109) [32], was cloned into the pET30a(+) vector at the BamHI/XhoI sites. The resulting fusion protein comprises the N-terminal histidine tag, S-tag, enterokinase cleavage site (E), and the capsid protein (N-HIS-S tag-E-capsid).

### 2.2. Protein Expression and Purification

The JEV capsid protein was expressed by autoinduction in terrific broth media without trace elements (Formedium) at 30 °C overnight in *E. coli* BL21(DE3). Cells were lysed in a high salt buffer (50 mM Tris pH 7.5, 1 M NaCl, 1 mg/mL lysozyme, 5 mM ethylenediaminetetraacetic acid (EDTA)). Protein was purified from the soluble fraction by immobilized nickel ion affinity (Ni-NTA) chromatography and dialyzed against a 25 mM Tris pH 7.6, 50 mM NaCl, 2 mM CaCl_2_ buffer overnight. The protein remained in this buffer throughout the following purification processes. The sample was then centrifuged at 16,000× *g* for 20 min at 4 °C to separate precipitation. The S-tag was cleaved by enterokinase (New England Biolabs, Ipswich, MA, USA) at 1:100 ratio enzyme to capsid protein at 4 °C overnight. Fusion tags were removed by filtration through Ni-NTA. Concentrated flow-through was subjected to gel filtration on a Superdex 75 10 × 300 mm size-exclusion chromatography column (GE Life Science, Boston, MA, USA). The peak at a retention volume of 13.5 mL was concentrated and used for crystallization.

### 2.3. Mass-Spectrometry Analysis

Purified capsid protein bands were excised from an SDS-PAGE gel and preserved in 20% ethanol. They were washed for 30 min twice with 50% acetonitrile and 0.2 M ammonium bicarbonate pH 8.9 and then dried in a rotary evaporator. The gel pieces were rehydrated in 2 M urea and 0.2 M ammonium bicarbonate pH 7.8 (Rehydration buffer RHB), containing 0.1 µg trypsin and incubated at 37 °C overnight. Excess RHB was then removed to a new 1.5 mL microfuge tube and peptides were extracted from the gel pieces with 60% acetonitrile and 0.1% trifluoroacetic acid (TFA). The total peptide extract was then concentrated to 10 µL in a rotary evaporator and then desalted using C18 (200 Å pore size silica resin) ZipTips (Milipore), according to the manufacturer’s instructions. Mass spectrometry (MS) analysis was performed using a MALDI-ToF instrument (Waters–Micromass), using a saturated solution of alpha-cyano-4 hydroxycinnaminic acid (CHCA) in 50% acetonitrile/0.1% trifluoroacetic acid. Samples were selected in the mass range of 850–2500 Da.

### 2.4. Protein Crystallization

Capsid protein at a concentration of ~6 mg/mL produced needle crystals in a 18% *v*/*v* 2-propanol, 0.1 M sodium citrate tribasic dihydrate pH 4.6–6.2, 16–18% *w*/*v* polyethylene glycol (PEG) 4000 reservoir solution. The crystals were flash frozen in a reservoir solution pH 5.6, containing 25% ethylene glycol.

### 2.5. Diffraction Experiment, Data Processing, and Model Building

X-ray data were collected at a cryogenic temperature at beamline I04 at Diamond Light Source, UK. Data was processed using Xia2 [33]. Crystals diffracted x-rays to 1.98 Å resolution. The protein structure was determined by molecular replacement using the structure of the WNV capsid protein (Protein Data Bank identifier: 1SFK, 63% sequence identity) as a starting model. Automated model building was performed with Buccaneer [34]. The structure was refined with REFMAC5 [35] and built in COOT [36], within the CCP4 program suite. The first 25 residues at the N-terminus are not visible in the electron density map. Data collection and refinement statistics are shown in Table S1. The JEV capsid refinement statistics of the Ramachandran plot are 100% favored and 0% outliers. The MolProbity score is 1.03. Superposition was performed by MatchMaker in Chimera with the default setting.

### 2.6. Data Deposition

The atomic coordinates and structure factors for the JEV capsid have been deposited in the Protein Data Bank, www.pdb.org with PDB ID 5OW2.

## 3. Results and Discussion

### 3.1. The JEV Capsid Protein N-terminus is Prone to Proteolytic Cleavage

**Figure 1:**
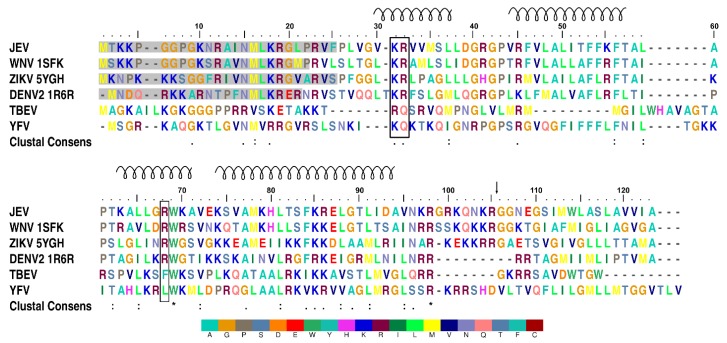
Multiple sequence alignment of flavivirus capsid proteins. Positively charged residues, arginine (R) and lysine (K), accumulate at the N- and C-termini. Lysine 31, arginine 32, and arginine 68 are highlighted with a black box. The spiral above the sequence indicates the α-helical secondary structure and numbering, which is based on the JEV capsid protein. Residues that are not visible in protein structures are shaded in grey. The black arrow marks the natural NS3 protease cleavage site. An asterisk indicates a fully conserved residue. A colon indicates conservation between groups of strongly similar properties. A period indicates conservation between groups of weakly similar properties. The alignment was produced with MUSCLE [40]. Color is given to each residue with the legend below.

Multiple JEV capsid protein species were observed on the purification of the recombinant protein indicating protein degradation. Mass spectrometry confirmed that two different size bands observed by SDS-PAGE were both N-terminally truncated the JEV capsid protein (Figure S1). Three flavivirus capsid structures from the DENV, ZIKV, and WNV have been solved previously using both NMR [37] and x-ray crystallography [15,38], respectively. One common feature of the protein is the unstable N-terminus. The first 20 residues of the DENV capsid NMR structure (PDB: 1R6R) are conformationally plastic, and the WNV capsid structure (PDB: 1SFK) is stable only from residue 23. Similarly, electron density for the first 25 residues from the JEV C protein N-terminus is not visible in our crystal structure due to cleavage during protein purification (Figure 1). *E. coli* cysteine proteases may act in a fashion similar to human Cathepsin L, which is known to cleave the C protein between the highly conserved residues Lys18 and Arg19 [39].

### 3.2. JEV Capsid Protein Structure

**Figure 2:**
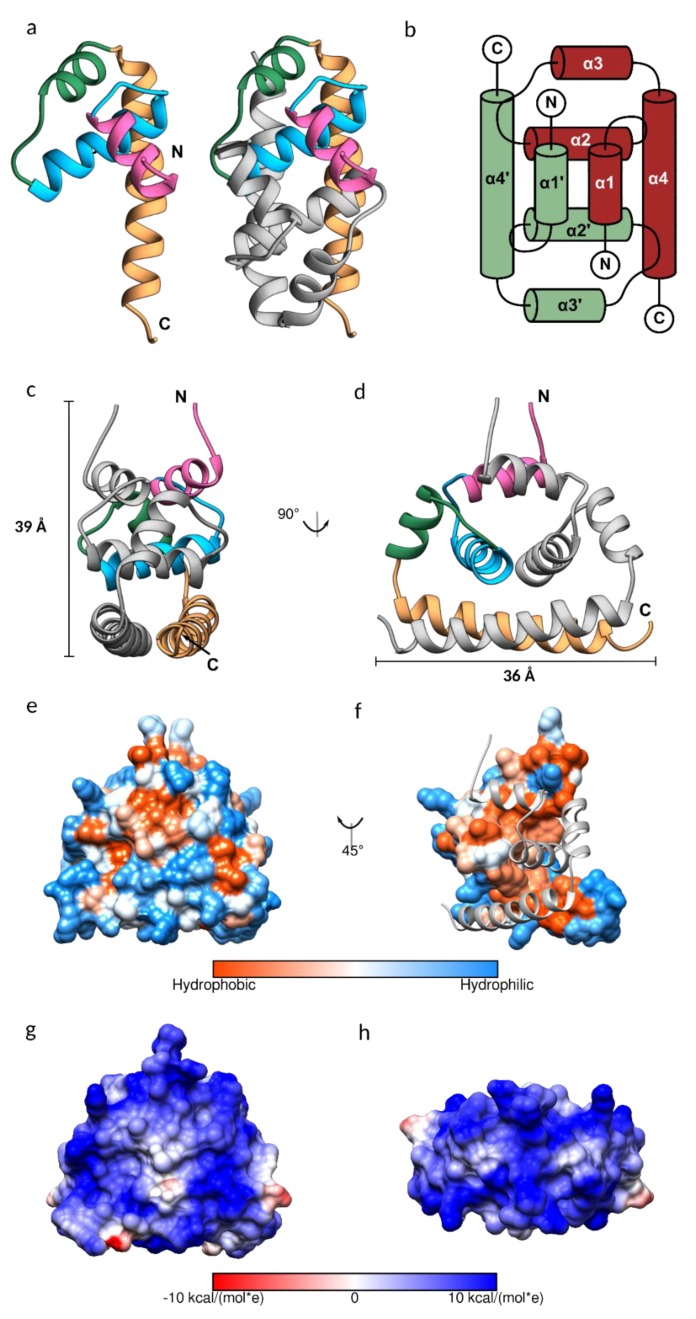
The structure of the Japanese encephalitis virus (JEV) capsid protein. (**a**) Ribbon model of the JEV capsid monomer, colored by the 1–4 α helices in pink, blue, green, and sandy brown, respectively. The dimeric assembly is also shown with the second subunit in grey. (**b**) Topology diagram of the JEV capsid dimer. The α-helices of one subunit are indicated with an apostrophe symbol. (**c,d**) Side view of the JEV capsid dimer with the dimension of 3.9 × 3.6 nm. (**e,f**) Hydrophobic surface colored on the Kyte–Doolittle scale. (**e**) Hydrophobic surface of the dimer. (**f**) Hydrophobic surface of the monomer with the opposing subunit is shown as a ribbon model. (**g,h**) Coulombic surface coloring of the capsid dimer side view and top view, respectively. The surface shows a symmetric pattern consistent with the homodimers. Electrostatic surface potential was calculated according to Coulomb’s law, with thresholds ±10 kcal/mol*e.

The purified JEV capsid protein, whose size was reduced to less than 15 kDa, was crystallized, and the solved structure has a visible electron density for residues 26–98. Each monomer of the JEV capsid protein is composed of four helices: α1 (amino acid 29–38), α2 (44–57), α3 (63–70), and the longest α4 (74–96), connected by short loops (Figure 2a–d). The protein contains substantially more positively charged than negatively charged amino acids (theoretical isoelectric point 12.6), which are distributed throughout the primary sequence but cluster at the N- and C-termini (Figure 1).

As is the case for the DENV, WNV, and ZIKV capsid proteins, the JEV capsid protein is a dimer, created by anti-parallel pairing of the α1-α1’, α2-α2’, and α4-α4’ helices, connected by extensive hydrophobic interactions, hydrogen bonds, and salt bridges (Tables S2–S4, Figure S2). The dimer is also stabilized by hydrogen bonds between α2 and α4 helixes. The JEV capsid dimer is connected by 13 hydrogen bonds, similar to the ZIKV capsid, with an average distance of 3.06 Å and 2.87 Å, respectively. The DENV capsid has no interaction between α1-α1’ and has six interface hydrogen bonds, whilst the WNV capsid interface surprisingly contains only three hydrogen bonds. Almost half of the amino acids of the JEV capsid, of which 41 are residues from the first subunit and 43 are residues from the second subunit, are involved in creating the hydrophobic dimer interface (Table S4, Figure 2f). A total of 16 of these are conserved among flavivirus capsid proteins (Table S4, scores 7 to 9). The large hydrophobic patch on the monomer surface that mediates dimerization is concealed after protein dimerization and the rest of the surface is hydrophilic (Figure 2e,f). This leads us to the conclusion that the capsid protein may prefer the dimeric arrangement. Furthermore, in our study, the soluble JEV capsid was efficiently isolated from bacterial cells, only in a high salt buffer, similar to the isolation of the tick-borne encephalitis virus (TBEV) capsid [41]. The high salt concentration in the lysis buffer may facilitate stable dimer formation.

It is worth noting that the capsid dimer has a coiled-coil-like structure formed by α4-α4’ helices, similar to a leucine zipper type DNA-binding protein (ZIP) (Figure 2a,c,d) [42]. The ZIP motif is labelled a–d, Ha-Pb-Pc-Hd-Ce-Pf-Cg, where H is the hydrophobic (leucine at every d position), P is polar, and C is charged. The JEV α4, consisting of only two of the repeated leucine residues, 78MaKbHcLdTeSfFgKaRbEcLdGeTfLg91, is shorter than the typical leucine zipper proteins and does not follow the heptad repeat pattern exactly. However, it has several hydrophobic residues forming a hydrophobic contact strip with α4’ from another subunit (Figure 1).

The capsid protein is known to form a spherical core enclosing the viral genome [3]. While tetrameric and hexameric forms were observed in the WNV and ZIKV capsid structures, respectively [15,38], they are absent from the JEV structure presented here, as was the case with the DENV C protein NMR structure. Cryo-EM structural studies of mature flaviviruses showed that the nucleocapsid density was low (~25–50%) compared with the envelope [2,43], indicating that the C protein assembly for forming the nucleocapsid is poorly ordered and is different from the icosahedral external shell [2,3,5,43,44], reflecting random interactions between the capsid and RNA [3,43]. However, a partially ordered dimeric capsid protein structure was observed in the immature ZIKV [45], suggesting a reorganization of the nucleocapsid in the virus life cycle. At neutral pH, the dimer net charge is +19 and the electrostatic surface map indicates an entirely electropositive surface with a symmetric distribution pattern (Figure 2g,h), which should repel neighboring capsid dimers, contradicting the dimer building block notion of nucleocapsid assembly. The lack of any higher order assembly may be due to the conformation of the protein. Protein assembly likely occurs after conformational change. Moreover, as virus assembly is a complex process and occurs with coordinating factors, an in vitro experiment that contains only capsid protein might not favor the capsid protein assembly process. An addition of interacting molecules is possibly required; for example, capsid-like particles were successfully produced from a dimeric capsid, isolated from the TBEV virions, incubated with viral RNA [41].

### 3.3. Conformational Plasticity of α-helix-1

**Figure 3:**
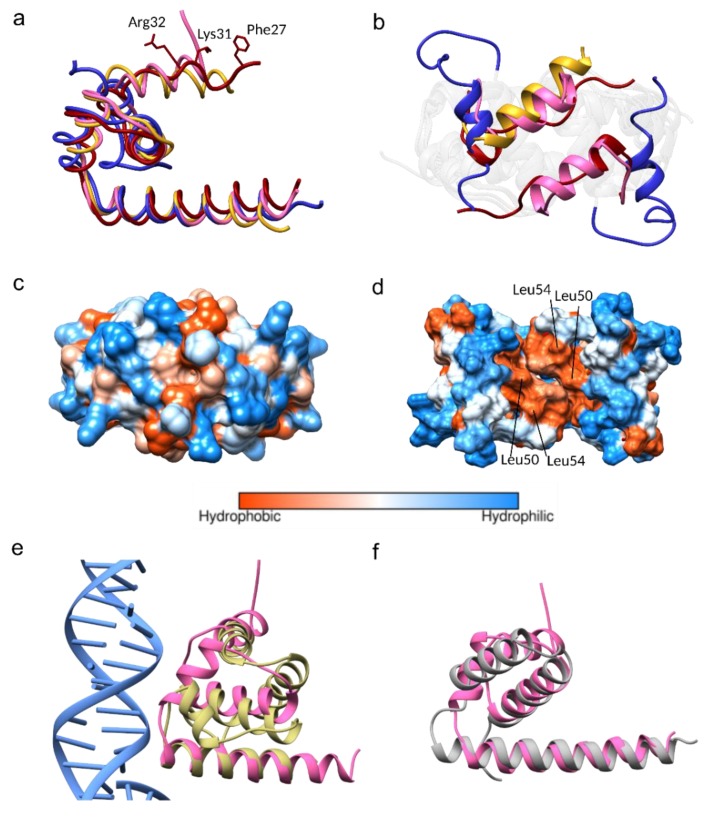
Comparison of the JEV, West Nile virus (WNV), dengue virus (DENV), and Zika virus (ZIKV) capsid proteins. (**a**) Superimposition of monomers of the WNV capsid (PDB: 1SFK) in gold, the DENV capsid (1R6R) in blue, and the ZIKV in red (5YGH) to the JEV capsid in pink. (**b**) Superposition of the α1-α1’ of the WNV, DENV, ZIKV, and JEV from the top view. Note that helix-1 in chain B of the WNV capsid is missing. (**c**) Hydrophobic surface of the JEV capsid showing the closed position and (**d**) similar view of the DENV capsid showing the opened position. (**e**,**f**) Structural homolog superimposition (**e**) The JEV capsid in pink is superimposed on transcription factor IIB (TFIIB) subunit of the transcription initiation complex (PDB 5FYW) and (**f**) human CNOT1 (PDB 4CQO).

The JEV capsid gene is the least conserved flavivirus capsid encoding gene (Figure 1). Despite that, the JEV capsid structure shares a similar dimeric state to the DENV, WNV, and ZIKV capsid structures, with Cα RMSD 1.063 Å, 0.896 Å, and 0.870 Å, respectively, except for helix-1, which is oriented in a different position (Figure 3a,b). The α1-α1’ helices of the JEV, WNV, and ZIKV are found on top of the α2-α2’ helices and protect the hydrophobic surface of α2-α2’, thereby forming a closed conformation. The DENV α1-helix is not paired with α1’ and moves aside perpendicularly to form an open conformation. This movement exposes and allows solvent to access the hydrophobic patch on the α2-α2’ surface, facilitating a hydrophobic interaction (Figure 3a–d) [18,19]. Without paring of the α1, the open dimeric form of the DENV has the least tight association, with a total buried surface area of 3037 Å2 compared to that of the WNV (3433 Å2), JEV (3905 Å2), and ZIKV (4658 Å2). The ZIKV capsid has tighter interaction, mainly due to the interaction mediated by the unstructured pre-α1 [15]. The N-terminus of this unique, long pre-α1 loop stabilizes the dimer by interacting with α2 and α3 of another dimer subunit. Similar to the ZIKV, the WNV capsid N-terminal residues make contacts beneath α2 and α3 to secure the dimer, while the N-terminus of the JEV capsid α1 is unstructured and lacks similar interactions. If a more complete N-terminal structure of the JEV capsid could be obtained, similar dimer reinforcing contacts would be expected. Determination of open conformation capsid structures by NMR may allow more flexibility than can be found in crystal structures, but it does not explain the disorder in the WNV capsid structure where the helix-1 of chain B and H are missing. Thus, flexibility is an inherent characteristic of the N-terminus itself.

Both of the α2 hydrophobic regions (Leu50 and Leu54) (Figure 3d) and the N-terminus of the DENV capsid protein have been suggested to interact with LDs [15,19,46]. The N-terminal interacting residues, Phe27, Lys31, and Arg32, were also identified from the ZIKV capsid structural study (Figure 3a) [15]. Comparable α2 hydrophobic properties and the conservation of Lys31 and Arg32 among the DENV, WNV, ZIKV, and JEV (Figure 1), indicate that similar LD interactions might apply to the JEV and WNV. Seemingly, either the open or closed capsid conformation makes interactions with LDs, and the conversion from the closed to the open conformation may be necessary for the α2-2’ membrane interaction.

Arg68, a conserved residue among the DENV, WNV, ZIKV, and JEV (Figure 1), located at the α3, is one of the residues responsible for side-chain packing of the 3-helix core and α1-α3 bundle in the open form of the DENV capsid structure (Figure S3) [37]. It is possible that Arg68 is the latch for the α1helix in the capsid open conformation. Arg68 is not conserved in yellow fever virus (YFV) and TBEV, which may not be necessary, as YFV and TBEV were predicted to contain only 3 helices, lacking α1 [47].

In this study, we crystallized the JEV capsid at pH 4.6, 5.6, and 6.2, each in similar crystal form with identical protein conformation. The DENV capsid protein, showing a distinct open conformation, was dissolved in buffer pH 6 [37], while WNV and ZIKV capsid crystals, which both displayed a closed conformation, were grown at pH 10.5 [38] and 5.6 [15], respectively. The factor that triggers conformational change of the capsid protein is unclear but it is clearly not pH related.

N-terminus flexibility may relate to the RNA binding capacity observed in the WNV capsid, where the first 32 residues bind to both the 5’ and 3’ untranslated region (UTR) of the WNV mRNA [13]. ST-148, a small molecule inhibitor, was reported to inhibit the DENV replication through an interaction at the α1 region of the capsid protein [48]. In an independent study, the N-terminus was shown to be important for viral propagation and the function is driven by its basic character [10].

### 3.4. Homologous Protein Superposition

A structural homolog search was performed by Dalilite v.3 [49]. Beyond the flavivirus proteins, the JEV capsid resembles the transcription factor IIB (TFIIB) subunit of the yeast polymerase II transcription initiation complex (PDB code 5FYW; Z= 4.5, RMSD= 2.9) and the human CCR4-NOT transcription complex subunit 1 (CNOT1) (4CQO; Z= 4.4, RMSD= 3.0) (Figure 3e,f). Both complexes have gene regulatory roles mediated by nucleic acid interactions. One monomer of the JEV capsid (α1–α4) could be aligned to the DNA interacting subunit of the TFIIB (residues 124–217), with α4-helix lying closest to the DNA fragment (Figure 3e). The monomer of the JEV capsid (α2–α4) aligns to the N-terminal of CNOT1 (residues 1842–1921) (Figure 3f). We note that only the monomer of the C protein could align to homologous proteins, in agreement with the findings of the WNV capsid structural study [9,38]. While other evidence supports the notion that the capsid dimer is the building block of the nucleocapsid [3,41], our homolog search suggested the alternative possibility of interaction with the monomeric state.

In addition, a capsid-RNA interaction may occur in the monomeric state because the capsid protein was isolated in a detergent-containing buffer in the study by Khromykh et al. [13], which may mask the dimer interface hydrophobic patch and allow the protein to form a stable monomer. In this study, we also found that the capsid protein is partially soluble in the buffer, containing detergent (Figure S4). Moreover, capsid-RNA binding was inhibited by high salt concentration (800 mM NaCl for full-length capsid) [13], possibly due to charge neutralization by salts, leading to RNA dissociation from protein [9]. However, if salt promotes dimer formation, it is probable, therefore, that RNA association is opposed by the dimer assembly. From several flavivirus capsid protein studies, the dimer appears to be the stable form. Nevertheless, robust evidence showing the nucleocapsid oligomeric building block is lacking. As mentioned above, the capsid protein may be highly dynamic, in that both monomeric and dimeric forms are functional but required for different tasks. Moreover, rearrangement of the protein may occur several times at different stages in virus maturation. However, while interesting, a functional role for the capsid monomer is currently lacking proof.

## 4. Conclusions

Capsid proteins form an inner shell to enclose a virus genome. They are multifunctional and essential for the virus life cycle. The JEV capsid protein has a helix-rich structure and forms a stable homodimer similar to other flaviviruses. The capsid N-terminus is unstable and the α-helix-1 is flexible, forming a closed conformation in the JEV. The α4-4’ site on the dimeric interface could be a potential viral genome RNA interaction site, due to its coiled-coil-like structure. In contrast, the monomer of the JEV capsid protein shows structural similarity to nucleic acid binding proteins. This suggests that capsid protein is highly dynamic. This is in agreement with rearrangement during virus maturation. Our findings begin to elucidate the mystery of capsid assembly and functional interaction. This understanding may provide an approach to developing treatments for flavivirus infections.

## Supplementary Materials

The following are available online at https://www.mdpi.com/1999-4915/11/7/623/s1, Figure S1: Mass-spectrometry analysis of the JEV capsid protein; Figure S2: Hydrogen bonds at the JEV capsid dimer interface; Figure S3: The DENV capsid α1-α3 bundle; Figure S4: The JEV capsid protein lysis buffer screening; Table S1: Data collection and refinement statistics; Table S2: Hydrogen bonds between the JEV capsid dimer interfacing residues and the distance; Table S3: Salt bridges between the JEV capsid dimer interfacing residues; Table S4: The JEV capsid dimer interfacing residues reported with accessible surface area (ASA) and buried surface area (BSA), solvation energy effect (ΔG) and conservation score.
