# Supplementary material for: Crystal Structure of the Japanese Encephalitis Virus Capsid Protein
Source: Viruses. 2019 Jul 6;11(7):623. doi: 10.3390/v11070623 (PMC6669762; doi:10.3390/v11070623)
Supplement: Supplementary file 1 [file viruses-11-00623-s001.pdf]

viruses

MDPI

Supplementary Materials

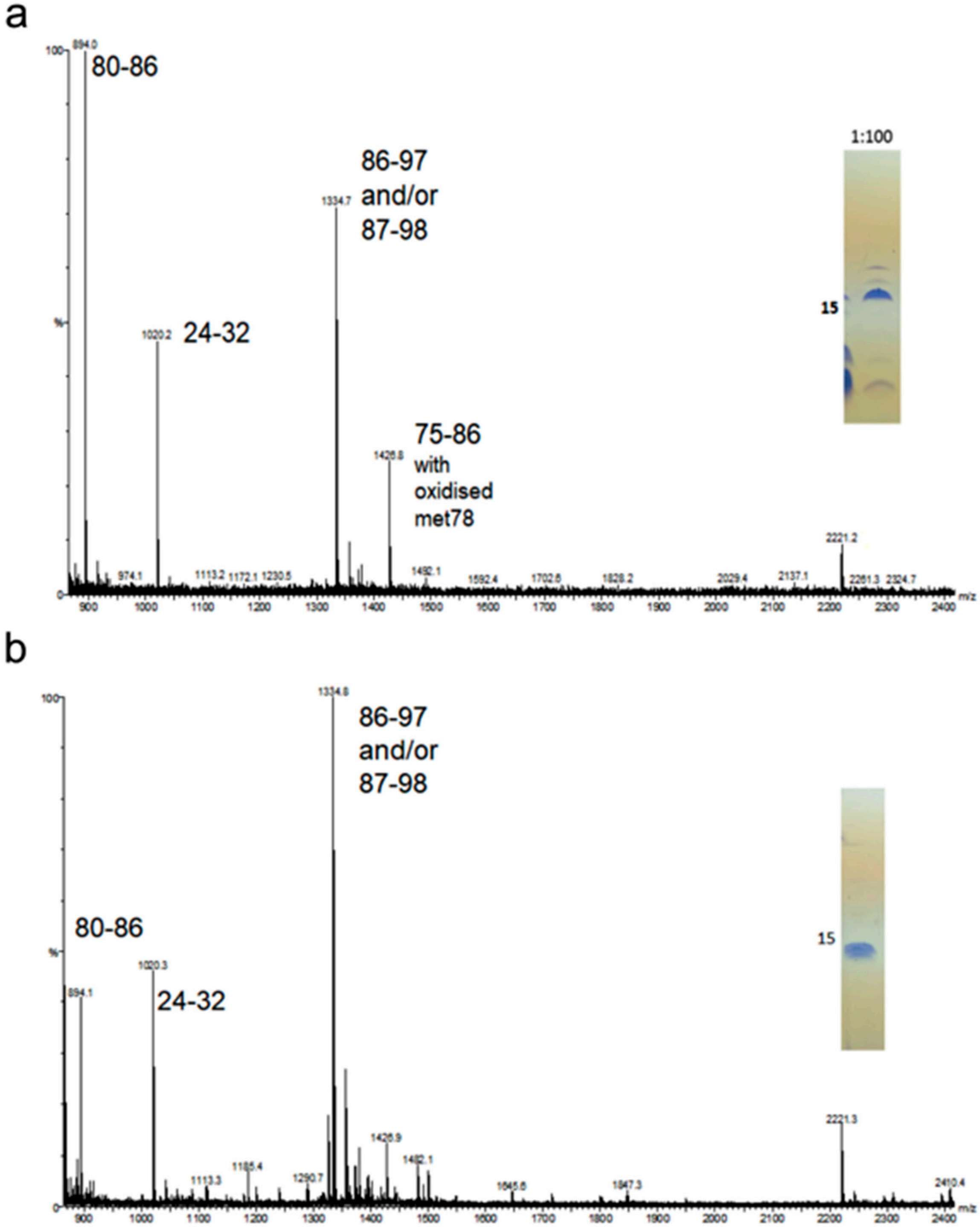

Figure S1. Mass-spectrometry analysis of JEV capsid protein. MS analysis of digested (**a**) and purified capsid (**b**). SDS-PAGE gels of the samples used for MS analysis are shown. Residue numbers are indicated next to the peaks.

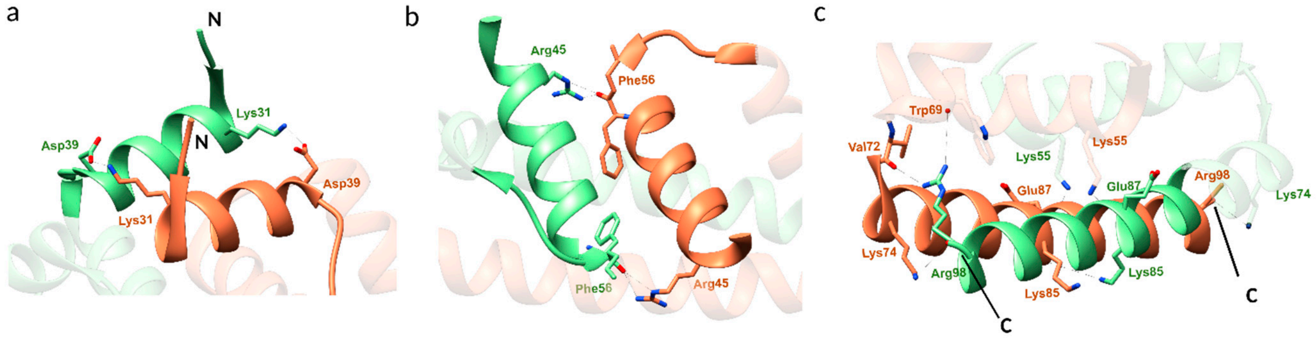

Figure S2. Hydrogen bonds at the JEV capsid dimer interface. Hydrogen bonds at the $\alpha1$-$\alpha1'$, $\alpha2$-$\alpha2'$, and $\alpha3$-$\alpha3'$ are shown with dashed lines in figure (**a**), (**b**), and (**c**), respectively. One monomer is in orange. Another is in green.

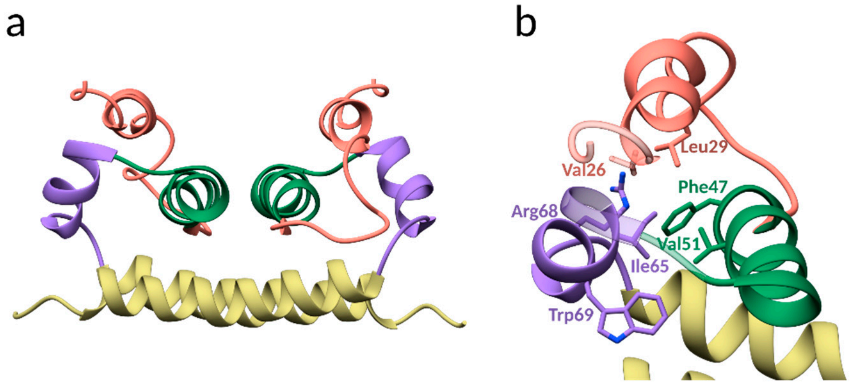

Figure S3. DENV capsid $\alpha1$-$\alpha3$ bundle. Residues invlove in side chain packing of DENV (PDB: 1R6R) capsid $\alpha1$-$\alpha3$ bundle are labeled. Each monomer coloured by the 1-4 α helices in orange, green, purple, and yellow, respectively.

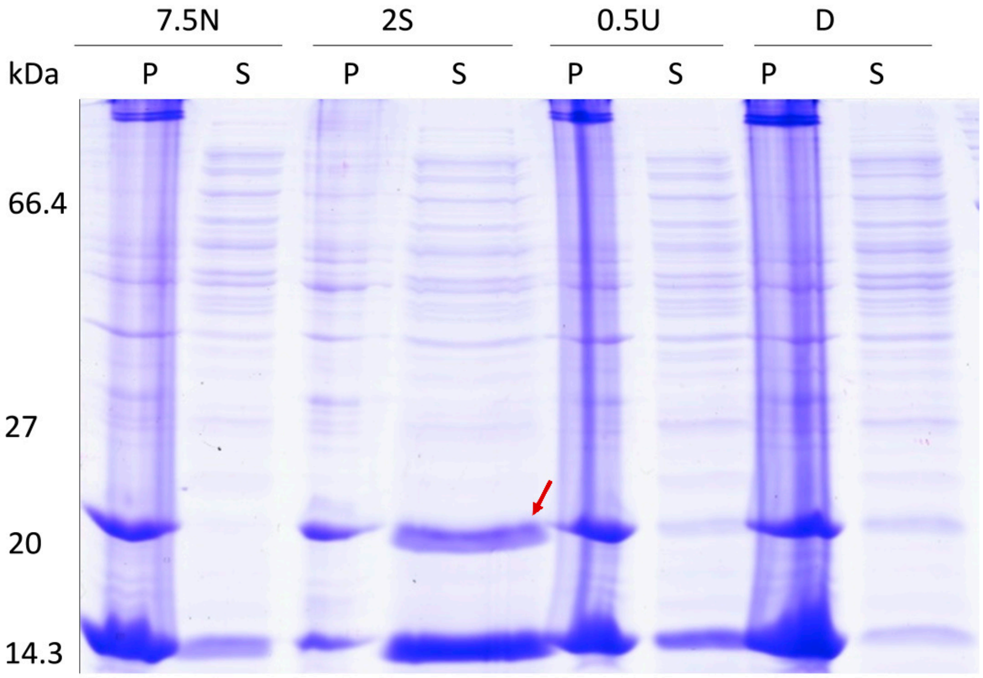

Figure S4. JEV capsid protein lysis buffer screening. Lysis buffer screening demonstrated that capsid protein is soluble in 2 M salt buffer indicating by red arrow and partially soluble in buffer containing urea and detergent. Pellet fractions and suspension fractions were labelled P and S, respectively. Four lysis buffers were the buffer added with the following additives: no additive (7.5N), 2 M NaCl (2S), 0.5 M urea (0.5U), and 0.2% Triton X 100 (D).

Table S1. Data collection and refinement statistics.

	**JEV Capsid**
**Data Collection**	
Space group	P212121
Cell dimensions	
a, *b*, *c* (Å)	46.31, 49.78, 68.25
α,β,γ(°)	90, 90, 90
Resolution (Å)	38.32-1.98(2.03-1.98)*
R_{merge}	0.058(0.862)
R_{pim}	0.044(0.686)
I / σ*I*	12.1(1.4)
CC half	0.999(0.551)
Completeness (%)	99.6(99)
Redundancy	4.5(4.3)
**Refinement**	
Resolution (Å)	38.32-1.98
No. reflections	10871
R_{work} / R_{free}	0.188/0.237
No. atoms	1229
Protein	1144
Water	56
Ethylene glycol	16
Citrate ion	13
B-factors	
Protein	42.77
Water	53.26
Ethylene glycol	52.91
2-propanol	-
Citrate ion	66.22
R.m.s. deviations	
Bond lengths (Å)	0.014
Bond angles (°)	1.428

Table S2. Hydrogen bonds between JEV capsid dimer interfacing residues and the distance.

Number	Structure 1	Distance (Å)[1]	Structure 2
1	Lys 31 [NZ]	2.64	Asp 39 [OD1][2]
2	Arg 45 [NE]	3.11	Phe 56 [O]
3	Arg 45 [NH2]	3.13	Phe 56 [O]
4	Arg 98 [NH2]	3.36	Trp 69 [O]
5	Arg 98 [NH1}	2.94	Val 72 [O]
6	Lys 85 [NZ]	3.87	Lys 85 [O]
7	Lys 55 [NZ}	3.03	Glu 87 [O}
8	Lys 74 [NZ]	2.77	Arg 98 [O]
9	Asp 39 [OD1]	2.69	Lys 31 [NZ]
10	Phe 56 [O]	3.16	Arg 45 [NH2]
11	Phe 56 [O]	3.07	Arg 45 [NE]
12	Glu 87 [O]	3.00	Lys 55 [NZ]
13	Arg 98 [O]	2.98	Lys 74 [NZ]

[1] Assembly analysis in the program PISA [50].

Table S3. Salt bridges between JEV capsid dimer interfacing residues.

Number	Structure 1	Distance (Å)[1]	Structure 2
1	Lys 31 [NZ]	2.64	Asp 39 [OD1]
2	Lys 74 [NZ]	2.77	Arg 98 [O]
3	Asp 39 [OD1]	2.69	Lys 31 [NZ]
4	Arg 98 [O]	2.98	Lys 74 [NZ]

[1] Assembly analysis in the program PISA.

Table S4. JEV capsid dimer interfacing residues reported with accessible (ASA) and buried surface area (BSA), solvation energy effect (ΔG) and conservation score.

(The values are reported from one monomer)

Number	Residue	ASA (Å²)	BSA (Å²)	ΔG (kcal/mol)	Conservation[1]
1	Leu27	83.65	21.41	0.34	1
2	Val30	117.35	93.60	1.5	2*
3	Lys31	144.46	90.81	0.19	8
4	Val33	69.18	18.08	0.29	3*
5	Val34	64.36	56.65	0.91	6*
6	Met35	100.45	54.43	1.29	1
7	Leu37	10.72	9.71	0.16	5*
8	Leu38	49.69	37.96	0.59	3*
9	Asp39	74.39	20.45	-0.36	2*
10	Arg45	84.22	18.52	-0.08	6*
11	Phe46	93.98	43.04	0.67	2*
12	Ala49	41.95	41.95	0.56	7
13	Leu50	24.80	24.80	0.38	2*
14	Ile51	19.52	7.19	0.12	5*
15	Thr52	28.95	28.56	0.17	5*
16	Phe53	92.37	91.73	1.47	7*
17	Phe54	22.78	16.86	0.27	5*
18	Lys55	164.75	92.62	-1.11	7*
19	Phe56	146.69	144.04	1.49	6*
20	Thr57	60.84	42.49	0.44	6*
21	Leu59	102.50	49.22	0.79	7*
22	Ala60	89.25	18.81	-0.10	5*
23	Thr62	52.30	27.04	0.43	8
24	Trp69	87.57	51.05	0.60	8
25	Lys70	155.76	6.41	0.02	
26	Lys72	25.04	11.51	-0.11	
27	Lys74	158.12	83.67	-0.29	6*
28	Ala77	16.40	16.40	0.26	9
29	Met78	106.53	38.83	0.97	7*
30	Leu81	56.97	56.80	0.77	8
31	Thr82	69.35	21.75	0.35	2*
32	Phe84	36.14	36.14	0.51	7*
33	Lys85	156.49	85.08	-0.30	7
34	Glu87	97.01	47.74	0.13	6*
35	Leu88	79.21	79.04	1.24	7
36	Gly89	30.81	14.57	0.21	5*
37	Thr90	93.35	9.88	0.16	4*
38	Leu91	108.63	90.54	1.44	7
39	Ile92	91.95	74.87	1.19	6*
40	Val95	102.29	87.78	1.23	4*
41	Asn96	114.80	45.24	-0.49	8
42	Lys97	165.54	0.48	0.01	3*
43	Arg98	130.94	29.07	0.03	9

[1] Amino acid conservation scores are given by Consurf. [51] (9 = conserved and 1 = variable) The following UniProtKB were submitted for conservation score calculation: W0LHC1, A0A1B2CW17, A0A0A7AAT7, W0LLY6, Q32ZE1, P12823, P14335, Q89277, P07720, and P27395.

- Below the confidence cut-off [51].

- **References**
-

- 50. Krissinel, E.; Henrick, K. Inference of macromolecular assemblies from crystalline state. *J. Mol. Biol.* **2007**, *372*, 774–797. doi:10.1016/j.jmb.2007.05.022.
- 51. Landau, M.; Mayrose, I.; Rosenberg, Y.; Glaser, F.; Martz, E.; Pupko, T.; Ben-Tal, N. ConSurf 2005: the projection of evolutionary conservation scores of residues on protein structures. *Nucleic Acids Res.* **2005**, 33, W299-302. doi:10.1093/nar/gki370.
